# Association of COVID-19 mortality with COVID-19 vaccination rates in Rhineland-Palatinate (Germany) from calendar week 1 to 20 in the year 2021: a registry-based analysis

**DOI:** 10.1007/s10654-021-00825-6

**Published:** 2021-12-12

**Authors:** Daniel Wollschläger, Emilio Gianicolo, Maria Blettner, Ruben Hamann, Nils Herm-Stapelberg, Melissa Schoeps

**Affiliations:** 1 Institute of Medical Biostatistics, Epidemiology and Informatics (IMBEI), University Medical Center of the Johannes Gutenberg-University Mainz, Langenbeckstraße 1, 55131 Mainz, Germany; 2 Institute of Clinical Physiology of the Italian National Research Council (IFC-CNR), Lecce, Italy; 3 Division of Vaccine Documentation, Cancer Registry Rhineland-Palatinate, Mainz, Germany

**Keywords:** COVID-19, Mortality, Vaccination, Real-world evidence

## Abstract

Vaccination is among the measures implemented by authorities to control the spread of the COVID-19 pandemic. However, real-world evidence of population-level effects of vaccination campaigns against COVID-19 are required to confirm that positive results from clinical trials translate into positive public health outcomes. Since the age group 80 + years is most at risk for severe COVID-19 disease progression, this group was prioritized during vaccine rollout in Germany. Based on comprehensive vaccination data from the German federal state of Rhineland-Palatinate for calendar week 1–20 in the year 2021, we calculated sex- and age-specific vaccination coverage. Furthermore, we calculated the proportion of weekly COVID-19 fatalities and reported SARS-CoV-2 infections formed by each age group. Vaccination coverage in the age group 80 + years increased to a level of 80% (men) and 75% (women). Increasing vaccination coverage coincided with a reduction in the age group’s proportion of COVID-19 fatalities. In multivariable logistic regression, vaccination coverage was associated both with a reduction in an age-group’s proportion of COVID-19 fatalities [odds ratio (OR) per 5 percentage points = 0.89, 95% confidence interval (CI) = 0.82–0.96, *p* = 0.0013] and of reported SARS-CoV-2 infections (OR per 5 percentage points = 0.82, 95% CI 0.76–0.88, *p* < 0.0001). The results are consistent with a protective effect afforded by the vaccination campaign against severe COVID-19 disease in the oldest age group.

**Supplementary Information:**

The online version contains supplementary material available at 10.1007/s10654-021-00825-6.

## Introduction

Excess mortality during the SARS-CoV-2 pandemic has been observed worldwide including Germany [1, 2]. Vaccination is among the measures implemented by authorities to reduce the disease burden of the COVID-19 pandemic. Clinical trials have demonstrated the efficacy of COVID-19 vaccines in preventing symptomatic and severe disease in a controlled environment [3]. Still, real-world evidence of the public health effects of vaccination campaigns is essential due to possible selection effects in trial participation, and due to the spread of new virus variants that were not present during trials [4, 5]. The level of protection afforded by vaccination in the elderly population is particularly important, as this age group is by far most at risk for severe COVID-19 disease progression and mortality [6]. Based on comprehensive, centrally recorded documentation of vaccinations during the first vaccine rollout in the German federal state of Rhineland-Palatinate, we investigated the association between age-specific vaccination coverage and the age-specific decline in COVID-19 fatalities and SARS-CoV-2 infections from calendar week 1–20 in the year 2021. We expected public health effects primarily in age group 80 + years since this age group was vaccinated first, and previously bore the largest burden of COVID-19 mortality.

## Materials and methods

### Data sources

The Rhineland-Palatinate Statistical Office provided population counts for the reference date December 31st, 2019 as well as weekly overall mortality counts from January to June 2021, both stratified by sex and age group (15–34, 35–59, 60–79, 80 + years).

The Robert Koch Institute published weekly counts of COVID-19 deaths and SARS-CoV-2 infections as reported according to the Infection Protection Act [7], both stratified by sex and age group (15–34, 35–59, 60–79, 80 + years). Deaths were assigned to the location of the reporting municipal health authority.

During the initial vaccine rollout in 2021, the Division of Vaccine Documentation of the Cancer Registry Rhineland-Palatinate was put in charge of documenting all individual vaccinations with a COVID-19 vaccine provided by the state. This included vaccinations in dedicated centers as well as those carried out by mobile teams for nursing home residents, and by some company doctors. For each vaccinated person, the data set included information on sex, age, location of residence, vaccination dates, and the status of partial versus full vaccination. Individuals were considered fully vaccinated at the date of their second shot, at the date of their first shot after recovery from a previous SARS-CoV-2 infection, or at the date of their first shot with a vaccine requiring only one dose.

### Statistics

Vaccination data was preprocessed to exclude out-of-state residents and implausible observations (Online Resource). To obtain the same aggregation level as for the remaining data, individual data on vaccinations was aggregated by calendar week, sex, and age group (15–34, 35–59, 60–79, 80 + years). The data set used for all analyses resulted from merging the aggregated data sets on the resident population, all-cause mortality, SARS-CoV-2 infections, COVID-19 fatalities, and vaccinations, respectively. The units of observation were formed by all 160 combinations of calendar week, sex, and age group (Online Resource).

The aggregated data set contained the vaccination coverage as the percentage of the population fully vaccinated per calendar week, sex, and age group. The same information was included for vaccination dates that were time-lagged with a period of 14 days to account for the delay until reaching full protection.

In each calendar week, the sex-specific proportion of COVID-19 fatalities and of reported SARS-CoV-2 infections formed by each age group was calculated.

The association between vaccination coverage and COVID-19 mortality was explored by plotting the time course of these two measures together: (1) vaccination coverage, defined as the proportion of the population either at least partially or fully vaccinated and (2) the proportion of COVID-19 fatalities formed by each age group. A multivariable binomial logistic regression model was used to examine the association between the time-lagged vaccination coverage and the sex-specific proportion of weekly COVID-19 fatalities formed by each age group while adjusting for sex and age group (Online Resource). A second model for the sex-specific proportion of reported weekly SARS-CoV-2 infections formed by each age group used the same covariates. As a sensitivity analysis to test whether associations were specific to COVID-19 endpoints, a third analogous model for all-cause mortality was fitted (Online Resource). We report adjusted odds ratios together with 95% confidence intervals and *p *values from Wald-tests based on heteroscedasticity and autocorrelation-consistent standard errors [8] to account for autocorrelation of the time series.

We evaluated data from calendar week 1 to 20 in 2021 because fewer than 10 COVID-19 deaths per week were reported in Rhineland-Palatinate thereafter. Furthermore, rollout of vaccinations in private practices that were not documented in the available database started to increase in calendar week 15, leading to potential underestimation of vaccination coverage. Data analysis was carried out in the statistical environment R [9].

## Results

### COVID-19

**Fig. 1:**
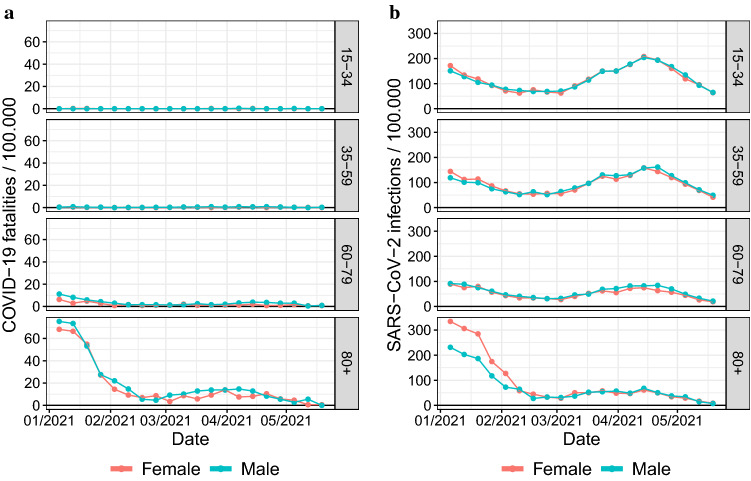
Reported sex- and age-specific counts per 100,000 residents for COVID-19 fatalities (**a**) and SARS-CoV-2 infections (2) from calendar week 1 to week 20 in 2021 in Rhineland-Palatinate, Germany

From calendar week 1 to 20 in 2021, there were 74,391 reported SARS-CoV-2 infections overall in Rhineland-Palatinate. In all age groups, infections dropped substantially from week 1 to 8 (Fig. 1). After school holidays around Easter from week 13 to 14, infections rose towards week 16 with higher increases in younger age groups but almost no change in age group 80 + years (Fig. 1). From week 1 to 20, the proportion of reported SARS-CoV-2 infections formed by age group 80 + years dropped from 16 to 1%.

**Fig. 2:**
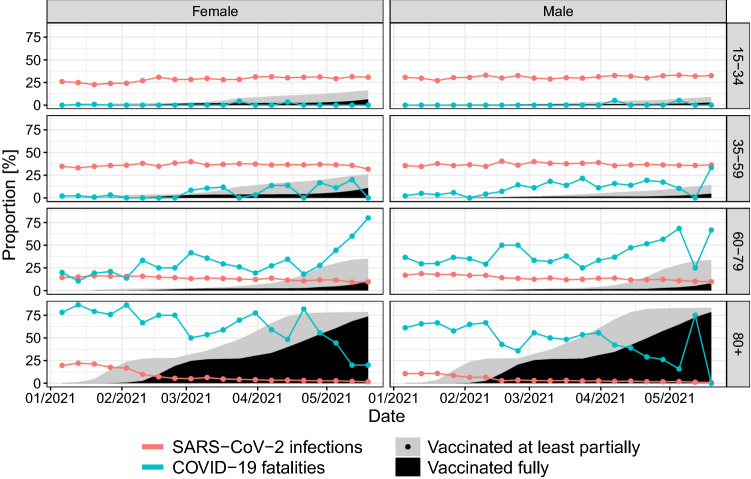
Age-specific time trends of the cumulative proportion vaccinated against COVID-19 at least partially or fully together with time trends of the proportion of COVID-19 fatalities and of reported SARS-CoV-2 infections formed by each age group from calendar week 1 to week 20 in 2021 in Rhineland-Palatinate, Germany

During the same period, there were 19,516 deaths overall in Rhineland-Palatinate with 11,594 (59.4%) deaths in the age group 80 + years and 6221 (31.9%) in the age group 60–79 years. There were 1554 COVID-19 fatalities, of which 999 (64.3%) were in the age group 80 + years and 456 (29.3%) in the age group 60–79 years (Figs. 1, 2). COVID-19 fatalities decreased from week 1 to week 7 in age groups 60–79 and 80 + years (Fig. 1). While 68–76% of COVID-19 fatalities were in the age group 80 + years until week 5, this proportion dropped to 9% in week 20 (Fig. 2). In calendar week 19, there were 5 COVID-19 deaths among women and 8 among men. Among men, 6/8 deaths (75%) were in the 80 + age group, causing the upward spike in the downward trend (Fig. 2).

### Vaccinations

From week 1 to 20 in 2021, the data set contained records for 1,054,339 individuals who received at least one shot, and for 578,088 individuals who received two shots. After applying exclusion criteria, 1,047,128 records of at least partially vaccinated individuals remained (Online Resource). Out of all fully vaccinated individuals, 9,901 (1.7%) only received one shot.

The time course of the vaccination activity was age- and sex-dependent. In age group 80 + years, the number of vaccinations increased sharply from week 2 onward and then began to decrease from week 19. The vaccination coverage in this age group reached 80% (men) and 75% (women) at week 20 (Fig. 2).

In the age group 60–79 years, the number of vaccinations increased from week 14 onward and reached a vaccination coverage of 15% at week 20 without relevant differences between men and women. In the lower age groups, a slight increase in the vaccination coverage was seen predominantly in women around week 7. Among 35–59 year-olds, the vaccination coverage reached 15% (women) and 7% (men) at week 20. Among 15–34 year-olds, it reached 9% (women) and 4% (men).

### Association of vaccination coverage with COVID-19 cases

Proportions of COVID-19 fatalities formed by age group 80 + years began to fall from calendar week 6 onward when vaccination coverage reached 26%. Exceptions to this trend among 80 + year-olds were observed in week 16 for women (18/22 COVID-19 fatalities) and week 19 for men (6/8 COVID-19 fatalities).

The vaccination coverage time-lagged by 14 days was associated with both a lower proportion of COVID-19 fatalities formed by an age group (adjusted odds ratio (OR) per 5 percentage points = 0.89, 95% confidence interval (CI) = 0.82–0.96, *p* = 0.001), and of reported SARS-CoV-2 infections (adjusted OR per 5 percentage points = 0.82, 95% CI 0.76–0.88, *p* < 0.0001) (Table 1). This means that an increase of 5 percentage points in vaccination coverage was associated with a 11% reduction (95% CI −18% to −4%) in the predicted odds for observing a COVID-19 fatality in an age group. For all-cause mortality, no such statistically significant association with vaccination coverage was observed (Online Resource).

**Table 1:** Adjusted odds ratios (OR), 95% confidence intervals (CI) and p-values (p) from multivariable logistic regression models for the sex-specific proportion of COVID-19 fatalities formed by each age group, and for the sex-specific proportion of reported SARS-CoV-2 infections formed by each age group

Variable	COVID-19 fatalities		SARS-CoV-2 infections	
	OR (95% CI)	*p*	OR (95% CI)	*p*
Female (ref)				
Male	1.04 (0.64–1.68)	0.88	0.96 (0.85–1.08)	0.47
Age 15–34	< 0.01 (< 0.01– < 0.01)	< 0.0001	2.89 (1.68–4.96)	0.0001
Age 35–59	0.03 (0.01–0.06)	< 0.0001	4.04 (2.38–6.85)	< 0.0001
Age 60–79	0.17 (0.09–0.31	< 0.0001	1.09 (0.63–1.88)	0.77
Age 80 + (ref)				
5% Points vaccination coverage	0.89 (0.82–0.96)	0.001	0.82 (0.76–0.88)	< 0.0001

## Discussion

The uptake of state-wide COVID-19 vaccinations in the age group 80 + years followed the beginning decline in reported SARS-CoV-2 infections in all age groups after high levels during the period from November 2020 to January 2021 [7]. Notably, SARS-CoV-2 infections fell in all age groups toward calendar week 6, even though vaccination coverage was close to zero except for 80 + year-olds. After observing excess mortality in November and December 2020, overall mortality rates fell markedly from calendar week 1 to week 6 [2]. A decline in COVID-19 fatalities toward week 6 was observed not only in age group 80 + years but also for 60–79 year-olds, before this age group was vaccinated.

The rollout of COVID-19 vaccination in Rhineland-Palatinate in the beginning of 2021 thus coincided with declining levels of all-cause mortality rates, and with the end of the second pandemic wave. These independent but parallel time trends made it difficult to directly evaluate the effectiveness of the vaccination campaign in a statistical model for COVID-19 mortality rates (Online Resource). However, because of the initial scarcity of the vaccine and prioritization of the age group 80 + years, the temporal trends in vaccination coverage were strongly age-dependent, such that their differential pattern afforded examination for associations with each age group’s share of COVID-19 fatalities.

While SARS-CoV-2 infections and COVID-19 fatalities diminished in all age groups toward week 6 in 2021, the fastest decline happened among 80 + year-olds. The resulting reduction in the proportion of COVID-19 fatalities formed by the 80 + age group coincided with its rapidly increasing vaccination coverage before other age groups started to catch up. These results were specific to COVID-19 endpoints, with no statistically significant association for all-cause mortality. On a country level, similar shifts in the age distribution of COVID-19 fatalities towards younger age groups have recently been described for countries with a vaccination policy that prioritized the elderly [10].

Our results are consistent with the assumption that COVID-19 vaccination has provided protection to the most vulnerable population from severe disease progression. There was a concurrent decline in the 80 + age group’s share of reported SARS-CoV-2 infections. Reported SARS-CoV-2 infections in the 80 + age group remained low even as infections in younger age groups increased again in April after school holidays around Easter. This observation is consistent with the assumption that the reduced COVID-19 mortality was mediated by fewer infections and not exclusively by reduced infection mortality. Another potential explanation is higher mobility and more social contacts among younger age groups during school holidays.

### Strengths and limitations

Until approximately calendar week 15 in 2021, vaccinations were performed almost exclusively with vaccines procured by the state of Rhineland-Palatinate. Since these vaccinations were all centrally documented, a strength of our study is the large dataset with detailed individual information and state-wide coverage. After calendar week 15, the use of vaccines from other supply chains increased. Such vaccinations were not centrally documented, limiting the validity of the study data set to the covered time period of 20 calendar weeks. A prerequisite for the bias-free calculation of the vaccination rate is that the population size was estimated with the same precision in all age groups. Given that recent validated end-of-year population counts were used, we assume that deviations were small and mostly random. Further limitations include that analyzing aggregated data carries the risk of ecological fallacy with spurious associations. Vaccinations administered by physicians in private practice were not included. Vaccinations performed in Rhineland-Palatinate of out-of-state residents were not considered. Estimates of COVID-19 mortality as well as of SARS-CoV-2 infections have inherent uncertainty due to heterogeneous testing strategy and capacity (Online Resource) as well as variability in cause-of-death coding. We here assume that these uncertainties did not change during the study period. Our results reflect public-health developments during early vaccine rollout, and are thus not informative with respect to long-term vaccine efficacy. The prioritization of population groups which determined access to vaccines was representative for Germany, but generalizability to regions with a different age structure and vaccination policy may be limited. If interpreted causally, the observed shift in the age distribution of COVID-19 fatalities would be a combined effect of the vaccination program which includes self-selection, vaccine-induced immunity, and possible behavioral changes with respect to risking exposure.

## Conclusions

A differential increase in vaccination coverage in the age group 80 + years was associated with a decrease in the proportion of COVID-19 fatalities and reported SARS-CoV-2 infections formed by this age group in Rhineland-Palatinate from calendar week 1 to week 20 in 2021. This result is consistent with the protective effect of COVID-19 vaccination against severe disease progression as demonstrated in clinical trials.

## Supplementary Information

Below is the link to the electronic supplementary material.

Supplementary file1 (PDF 168 kb)

## Data Availability

Data is available upon reasonable request.
