# Supplementary material for: Association of COVID-19 mortality with COVID-19 vaccination rates in Rhineland-Palatinate (Germany) from calendar week 1 to 20 in the year 2021: a registry-based analysis
Source: Eur J Epidemiol. 2021 Dec 12;36(12):1231–6. doi: 10.1007/s10654-021-00825-6 (PMC8665990; doi:10.1007/s10654-021-00825-6)
Supplement: Supplementary file 1 — Supplementary file1 (PDF 168 kb) [file 10654_2021_825_MOESM1_ESM.pdf]

# Association of COVID-19 mortality with COVID-19 vaccination rates in Rhineland-Palatinate (Germany) from calendar week 1 to 20 in the year 2021: A registry-based analysis

European Journal of Epidemiology - Online Resource

Daniel Wollschläger [1]
Emilio Gianicolo [1,2]
Maria Blettner [1]
Ruben Hamann [3]
Nils Herm-Stapelberg [3]
Melissa Schoeps [3]

[1] Institute of Medical Biostatistics, Epidemiology and Informatics, University Medical Center of the Johannes Gutenberg-University Mainz, Langenbeckstraße 1, 55131 Mainz, Germany. wollschlaeger@uni-mainz.de
[2] Institute of Clinical Physiology of the Italian National Research Council (IFC-CNR), Lecce, Italy.
[3] Division of Vaccine Documentation, Cancer Registry Rhineland-Palatinate, Mainz, Germany.

## 1 Data Preprocessing

In the vaccination data set, individuals residing outside of Rhineland-Palatinate were excluded to calculate sex- and age-specific vaccination coverage relative to the resident population. Individuals with missing information on sex or age were excluded for the same reason. In addition, implausible entries were excluded, for example when vaccination dates preceded vaccine availability, or when age exceeded 112 years.

Out of all 1054339 at least partially vaccinated individuals from calendar week 1 to 20 in 2021, 1359 (0.13%) were excluded due to implausible entries for vaccination date or age. Furthermore, 20291 (1.92%) out-of-state residents, 7206 (0.68%) individuals with unknown sex, and 5 (0.00047%) individuals with unknown age were excluded.

## 2 Rationale for the Choice of Regression Model

The initial vaccination rollout for age group 80+ years and health care workers in early 20201 coincided with the decline of the COVID-19 wave from December 2020 during which the elderly bore the major burden of COVID-19 mortality. Similarly, excess mortality during December 2020 was superseded by undermortality in the beginning of 2021 [1], possibly due to mortality displacement effects.

The incidental timing of the vaccine rollout thus created the risk of a spurious association between vaccination coverage and COVID-19 mortality rate in a Poisson regression model. Therefore, we did not analyze the absolute level of COVID-19 mortality rates as an endpoint. Instead, we modeled the shift in the age distribution of COVID-19 fatalities using a binomial logistic regression model which used the weekly sex-specific binomial proportion of COVID-19 fatalities in each age group as the endpoint. A similar analysis strategy has been used in a recent related publication, as well [2].

# 3 Sample Calculations

Table S1 lists the raw data used for all analyses. The following sections provide examples of how to perform calculations for the proportion vaccinated, and for the regression model endpoints.

## 3.1 Age-specific Proportion Vaccinated

In the study period, the residential population of females 80 years and older was estimated as 173095. In calendar week 5, there were 17089 completely vaccinated persons in that group. Therefore, the proportion vaccinated was 17089 / 173095 = 9.87%.

## 3.2 Proportion of COVID-19 Fatalities Formed by Each Age Group

In calendar week 5, there were 0, 0, 4, and 25 COVID-19 fatalities among females in age groups 15-34, 35-59, 60-79, and 80+ years, respectively. The sex-specific proportion of COVID-19 fatalities formed by females in the 80+ age group was thus 25/29 = 86%.

## 3.3 Proportion of Reported SARS-CoV-2 Infections Formed by Each Age Group

In calendar week 5, there were 317, 472, 205, and 220 reported SARS-CoV-2 infections among females in age groups 15-34, 35-59, 60-79, and 80+ years, respectively. The sex-specific proportion of reported SARS-CoV-2 infections formed by females in the 80+ age group was thus 220/1214 = 18%.

## 3.4 Regression Model

For the observational unit of calendar week 5, female sex, and age group 80+ years, the value of the endpoint "share of COVID-19 mortality" was 25/29 = 0.86. Corresponding covariate values were 0 (sex female = reference group), 0 (age group 80+ = reference group), and 1254/173095 = 0.72% (proportion vaccinated using a time lag of 14 days). This observation had a weight of 29 since there were 29 COVID-19 fatalities for females in that calendar week.

For the observational unit of calendar week 5, female sex, and age group 80+ years, the value of the endpoint "share of reported SARS-CoV-2 infections" was 220/1214 = 0.18. Corresponding covariate values were 0 (sex female = reference group), 0 (age group 80+ = reference group), and 1254/173095 = 0.72% (proportion vaccinated using a time lag of 14 days). This observation had a weight of 1214 since there were 1214 reported SARS-CoV-2 infections for females in that calendar week.

# 4 Timeline Non-Pharmaceutical Interventions

Table S2 provides an overview when individual non-pharmaceutical interventions were enacted during the study period in Rhineland-Palatinate, Germany [3].

# 5 Timeline Vaccination Program

Table S3 depicts the COVID-19 vaccination prioritization policy in Rhineland-Palatinate, Germany, from calendar week 1 to 20 in 2021. Due to limited supply of vaccines, groups who were most at risk for exposure to SARS-CoV-2 or those who were most vulnerable to COVID-19 disease were eligible first. This group included residents of age 80 and above, as well as health care workers with direct contact to possibly infected patients.

A recent publication [4] reported vaccination data from private practices. There, the proportion of vaccinated individuals age 60 and older declined from 71% in week 15 to 31% in week 20. Private practices therefore seemed to largely follow the priority policy.

# 6 Vaccines Used

Table S4 shows absolute and relative frequencies of vaccines used in the first shot received by individuals in Rhineland-Palatinate, Germany, from calendar week 1 to 20 in 2021, stratified by age group.

Table S5 shows absolute and relative frequencies of vaccines used in the second shot received by individuals in Rhineland-Palatinate, Germany, from calendar week 1 to 20 in 2021, stratified by age group.

# 7 Timeline Testing Capacity

Figure S1 shows the number of SARS-CoV-2 tests in Germany from calendar week 1 to 20 in 2021 as reported to the Robert Koch Institute [4].

Testing capacity increased during the first 6 calendar weeks and remained largely constant until week 20. Testing activity increased from week 9 to 12, then dropped in week 13 and 14 during school holidays around Easter. Testing activity subsequently increased towards week 16 before dropping toward week 20.

# 8 Sensitivity Analysis: Logistic Regression All-Cause Mortality

As a sensitivity analysis, a multivariable binomial logistic regression model was used to examine the association between the time-lagged vaccination coverage and the sex-specific proportion of weekly all-cause mortality formed by each age group while adjusting for sex and age group.

Adjusted odds ratios (OR) together with corresponding 95% confidence intervals (CI) and p-values are presented in Tab. S6. Results indicate that there was no statistically significant association between the age-specific share of all-cause mortality and vaccination coverage (adjusted OR = 0.996, 95% CI = [0.991; 1.001], p = 0.16).

# 9 Figures

Figure S1. SARS-CoV-2 tests and testing capacity in Germany from calendar week 1 to 20 in 2021 as reported to the Robert Koch Institute [5].

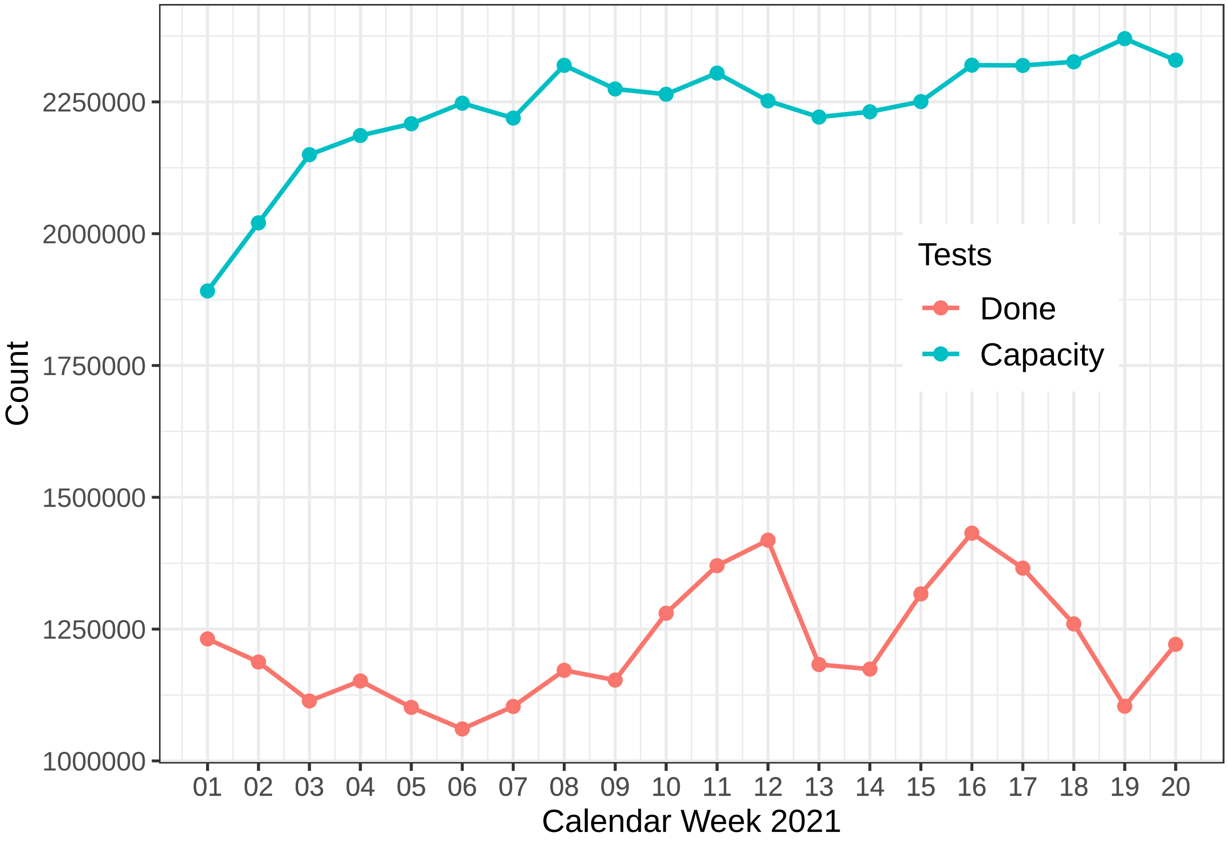

# 10 Tables

Table S1. Aggregated data set used for analyses. The units of observation are formed by all 160 combinations of calendar week in 2021 (1 to 20), sex (female, male), and age group (15-34, 35-59, 60-79, 80+ years) [6].

| Week | Sex | Age | Population | Vaccinated | Vaccinated Percent | Vaccinated Lag 14 | Vaccinated Lag 14 Percent | SARS-CoV-2 Incidence | SARS-CoV-2 Incidence all | SARS-CoV-2 Incidence share | COVID-19 Fatality | COVID-19 Fatality all | COVID-19 Fatality share |
|---|---|---|---|---|---|---|---|---|---|---|---|---|---|
| 1 | f | 15-34 | 445412 | 1 | 0.00 | 0 | 0.00 | 765 | 2946 | 0.26 | 0 | 151 | 0.00 |
| 1 | f | 35-59 | 709015 | 2 | 0.00 | 0 | 0.00 | 1022 | 2946 | 0.35 | 3 | 151 | 0.02 |
| 1 | f | 60-79 | 475567 | 0 | 0.00 | 0 | 0.00 | 424 | 2946 | 0.14 | 30 | 151 | 0.20 |
| 1 | f | 80+ | 173095 | 1 | 0.00 | 0 | 0.00 | 579 | 2946 | 0.20 | 118 | 151 | 0.78 |
| 1 | m | 15-34 | 482066 | 0 | 0.00 | 0 | 0.00 | 728 | 2373 | 0.31 | 0 | 134 | 0.00 |
| 1 | m | 35-59 | 709197 | 1 | 0.00 | 0 | 0.00 | 843 | 2373 | 0.36 | 3 | 134 | 0.02 |
| 1 | m | 60-79 | 438976 | 0 | 0.00 | 0 | 0.00 | 401 | 2373 | 0.17 | 49 | 134 | 0.37 |
| 1 | m | 80+ | 108937 | 0 | 0.00 | 0 | 0.00 | 252 | 2373 | 0.11 | 82 | 134 | 0.61 |
| 2 | f | 15-34 | 445412 | 36 | 0.01 | 0 | 0.00 | 598 | 2403 | 0.25 | 1 | 133 | 0.01 |
| 2 | f | 35-59 | 709015 | 156 | 0.02 | 0 | 0.00 | 796 | 2403 | 0.33 | 3 | 133 | 0.02 |
| 2 | f | 60-79 | 475567 | 86 | 0.02 | 0 | 0.00 | 358 | 2403 | 0.15 | 14 | 133 | 0.11 |
| 2 | f | 80+ | 173095 | 233 | 0.13 | 0 | 0.00 | 530 | 2403 | 0.22 | 115 | 133 | 0.86 |
| 2 | m | 15-34 | 482066 | 32 | 0.01 | 0 | 0.00 | 617 | 2078 | 0.30 | 0 | 122 | 0.00 |
| 2 | m | 35-59 | 709197 | 47 | 0.01 | 0 | 0.00 | 718 | 2078 | 0.35 | 6 | 122 | 0.05 |
| 2 | m | 60-79 | 438976 | 52 | 0.01 | 0 | 0.00 | 389 | 2078 | 0.19 | 36 | 122 | 0.30 |
| 2 | m | 80+ | 108937 | 62 | 0.06 | 0 | 0.00 | 221 | 2078 | 0.11 | 80 | 122 | 0.66 |
| 3 | f | 15-34 | 445412 | 299 | 0.07 | 1 | 0.00 | 528 | 2326 | 0.23 | 1 | 120 | 0.01 |
| 3 | f | 35-59 | 709015 | 925 | 0.13 | 2 | 0.00 | 807 | 2326 | 0.35 | 1 | 120 | 0.01 |
| 3 | f | 60-79 | 475567 | 521 | 0.11 | 0 | 0.00 | 380 | 2326 | 0.16 | 23 | 120 | 0.19 |
| 3 | f | 80+ | 173095 | 1254 | 0.72 | 1 | 0.00 | 493 | 2326 | 0.21 | 95 | 120 | 0.79 |
| 3 | m | 15-34 | 482066 | 165 | 0.03 | 0 | 0.00 | 507 | 1867 | 0.27 | 0 | 87 | 0.00 |
| 3 | m | 35-59 | 709197 | 305 | 0.04 | 1 | 0.00 | 705 | 1867 | 0.38 | 3 | 87 | 0.03 |
| 3 | m | 60-79 | 438976 | 322 | 0.07 | 0 | 0.00 | 329 | 1867 | 0.18 | 26 | 87 | 0.30 |
| 3 | m | 80+ | 108937 | 377 | 0.35 | 0 | 0.00 | 203 | 1867 | 0.11 | 58 | 87 | 0.67 |
| 4 | f | 15-34 | 445412 | 1757 | 0.39 | 36 | 0.01 | 413 | 1719 | 0.24 | 0 | 62 | 0.00 |
| 4 | f | 35-59 | 709015 | 3969 | 0.56 | 156 | 0.02 | 613 | 1719 | 0.36 | 2 | 62 | 0.03 |
| 4 | f | 60-79 | 475567 | 1417 | 0.30 | 86 | 0.02 | 272 | 1719 | 0.16 | 13 | 62 | 0.21 |
| 4 | f | 80+ | 173095 | 4390 | 2.54 | 233 | 0.13 | 302 | 1719 | 0.18 | 47 | 62 | 0.76 |

*(continued)*

| Week | Sex | Age | Population | Vaccinated | Vaccinated Percent | Vaccinated Lag 14 | Vaccinated Lag 14 Percent | SARS-CoV-2 Incidence | SARS-CoV-2 Incidence all | SARS-CoV-2 Incidence share | COVID-19 Fatality | COVID-19 Fatality all | COVID-19 Fatality share |
|---|---|---|---|---|---|---|---|---|---|---|---|---|---|
| 4 | m | 15-34 | 482066 | 987 | 0.20 | 32 | 0.01 | 454 | 1495 | 0.30 | 0 | 52 | 0.00 |
| 4 | m | 35-59 | 709197 | 1834 | 0.26 | 47 | 0.01 | 533 | 1495 | 0.36 | 3 | 52 | 0.06 |
| 4 | m | 60-79 | 438976 | 928 | 0.21 | 52 | 0.01 | 267 | 1495 | 0.18 | 19 | 52 | 0.37 |
| 4 | m | 80+ | 108937 | 2771 | 2.54 | 62 | 0.06 | 128 | 1495 | 0.09 | 30 | 52 | 0.58 |
| 5 | f | 15-34 | 445412 | 3995 | 0.90 | 299 | 0.07 | 317 | 1311 | 0.24 | 0 | 29 | 0.00 |
| 5 | f | 35-59 | 709015 | 9187 | 1.30 | 925 | 0.13 | 472 | 1311 | 0.36 | 0 | 29 | 0.00 |
| 5 | f | 60-79 | 475567 | 3468 | 0.73 | 521 | 0.11 | 205 | 1311 | 0.16 | 4 | 29 | 0.14 |
| 5 | f | 80+ | 173095 | 17089 | 9.87 | 1254 | 0.72 | 220 | 1311 | 0.17 | 25 | 29 | 0.86 |
| 5 | m | 15-34 | 482066 | 2239 | 0.46 | 165 | 0.03 | 374 | 1223 | 0.31 | 0 | 37 | 0.00 |
| 5 | m | 35-59 | 709197 | 4049 | 0.57 | 305 | 0.04 | 447 | 1223 | 0.37 | 0 | 37 | 0.00 |
| 5 | m | 60-79 | 438976 | 2206 | 0.50 | 322 | 0.07 | 204 | 1223 | 0.17 | 13 | 37 | 0.35 |
| 5 | m | 80+ | 108937 | 11840 | 10.87 | 377 | 0.35 | 79 | 1223 | 0.06 | 24 | 37 | 0.65 |
| 6 | f | 15-34 | 445412 | 6879 | 1.54 | 1757 | 0.39 | 278 | 1026 | 0.27 | 0 | 24 | 0.00 |
| 6 | f | 35-59 | 709015 | 16765 | 2.36 | 3969 | 0.56 | 390 | 1026 | 0.38 | 0 | 24 | 0.00 |
| 6 | f | 60-79 | 475567 | 6290 | 1.32 | 1417 | 0.30 | 163 | 1026 | 0.16 | 8 | 24 | 0.33 |
| 6 | f | 80+ | 173095 | 32917 | 19.02 | 4390 | 2.54 | 101 | 1026 | 0.10 | 16 | 24 | 0.67 |
| 6 | m | 15-34 | 482066 | 3769 | 0.78 | 987 | 0.20 | 352 | 1065 | 0.33 | 0 | 24 | 0.00 |
| 6 | m | 35-59 | 709197 | 6830 | 0.96 | 1834 | 0.26 | 369 | 1065 | 0.35 | 1 | 24 | 0.04 |
| 6 | m | 60-79 | 438976 | 3886 | 0.89 | 928 | 0.21 | 178 | 1065 | 0.17 | 7 | 24 | 0.29 |
| 6 | m | 80+ | 108937 | 22297 | 20.47 | 2771 | 2.54 | 70 | 1065 | 0.07 | 16 | 24 | 0.67 |
| 7 | f | 15-34 | 445412 | 8636 | 1.94 | 3995 | 0.90 | 336 | 1089 | 0.31 | 0 | 16 | 0.00 |
| 7 | f | 35-59 | 709015 | 22172 | 3.13 | 9187 | 1.30 | 379 | 1089 | 0.35 | 0 | 16 | 0.00 |
| 7 | f | 60-79 | 475567 | 8851 | 1.86 | 3468 | 0.73 | 161 | 1089 | 0.15 | 4 | 16 | 0.25 |
| 7 | f | 80+ | 173095 | 42493 | 24.55 | 17089 | 9.87 | 77 | 1089 | 0.07 | 12 | 16 | 0.75 |
| 7 | m | 15-34 | 482066 | 4648 | 0.96 | 2239 | 0.46 | 332 | 1108 | 0.30 | 0 | 14 | 0.00 |
| 7 | m | 35-59 | 709197 | 8705 | 1.23 | 4049 | 0.57 | 448 | 1108 | 0.40 | 1 | 14 | 0.07 |
| 7 | m | 60-79 | 438976 | 5460 | 1.24 | 2206 | 0.50 | 156 | 1108 | 0.14 | 7 | 14 | 0.50 |
| 7 | m | 80+ | 108937 | 27677 | 25.41 | 11840 | 10.87 | 30 | 1108 | 0.03 | 6 | 14 | 0.43 |
| 8 | f | 15-34 | 445412 | 9443 | 2.12 | 6879 | 1.54 | 297 | 1049 | 0.28 | 0 | 20 | 0.00 |
| 8 | f | 35-59 | 709015 | 24599 | 3.47 | 16765 | 2.36 | 403 | 1049 | 0.38 | 0 | 20 | 0.00 |
| 8 | f | 60-79 | 475567 | 10130 | 2.13 | 6290 | 1.32 | 149 | 1049 | 0.14 | 5 | 20 | 0.25 |
| 8 | f | 80+ | 173095 | 45679 | 26.39 | 32917 | 19.02 | 57 | 1049 | 0.05 | 15 | 20 | 0.75 |

*(continued)*

| Week | Sex | Age | Population | Vaccinated | Vaccinated Percent | Vaccinated Lag 14 | Vaccinated Lag 14 Percent | SARS-CoV-2 Incidence | SARS-CoV-2 Incidence all | SARS-CoV-2 Incidence share | COVID-19 Fatality | COVID-19 Fatality all | COVID-19 Fatality share |
|---|---|---|---|---|---|---|---|---|---|---|---|---|---|
| 8 | m | 15-34 | 482066 | 5058 | 1.05 | 3769 | 0.78 | 332 | 1017 | 0.33 | 0 | 14 | 0.00 |
| 8 | m | 35-59 | 709197 | 9452 | 1.33 | 6830 | 0.96 | 367 | 1017 | 0.36 | 2 | 14 | 0.14 |
| 8 | m | 60-79 | 438976 | 6261 | 1.43 | 3886 | 0.89 | 137 | 1017 | 0.13 | 7 | 14 | 0.50 |
| 8 | m | 80+ | 108937 | 28933 | 26.56 | 22297 | 20.47 | 36 | 1017 | 0.04 | 5 | 14 | 0.36 |
| 9 | f | 15-34 | 445412 | 9729 | 2.18 | 8636 | 1.94 | 279 | 984 | 0.28 | 0 | 12 | 0.00 |
| 9 | f | 35-59 | 709015 | 25329 | 3.57 | 22172 | 3.13 | 393 | 984 | 0.40 | 1 | 12 | 0.08 |
| 9 | f | 60-79 | 475567 | 10488 | 2.21 | 8851 | 1.86 | 130 | 984 | 0.13 | 5 | 12 | 0.42 |
| 9 | f | 80+ | 173095 | 46493 | 26.86 | 42493 | 24.55 | 49 | 984 | 0.05 | 6 | 12 | 0.50 |
| 9 | m | 15-34 | 482066 | 5199 | 1.08 | 4648 | 0.96 | 340 | 1139 | 0.30 | 0 | 18 | 0.00 |
| 9 | m | 35-59 | 709197 | 9714 | 1.37 | 8705 | 1.23 | 455 | 1139 | 0.40 | 2 | 18 | 0.11 |
| 9 | m | 60-79 | 438976 | 6489 | 1.48 | 5460 | 1.24 | 142 | 1139 | 0.12 | 6 | 18 | 0.33 |
| 9 | m | 80+ | 108937 | 29244 | 26.84 | 27677 | 25.41 | 34 | 1139 | 0.03 | 10 | 18 | 0.56 |
| 10 | f | 15-34 | 445412 | 9980 | 2.24 | 9443 | 2.12 | 405 | 1376 | 0.29 | 0 | 28 | 0.00 |
| 10 | f | 35-59 | 709015 | 25721 | 3.63 | 24599 | 3.47 | 498 | 1376 | 0.36 | 3 | 28 | 0.11 |
| 10 | f | 60-79 | 475567 | 10593 | 2.23 | 10130 | 2.13 | 190 | 1376 | 0.14 | 10 | 28 | 0.36 |
| 10 | f | 80+ | 173095 | 46861 | 27.07 | 45679 | 26.39 | 87 | 1376 | 0.06 | 15 | 28 | 0.54 |
| 10 | m | 15-34 | 482066 | 5311 | 1.10 | 5058 | 1.05 | 420 | 1454 | 0.29 | 0 | 22 | 0.00 |
| 10 | m | 35-59 | 709197 | 9910 | 1.40 | 9452 | 1.33 | 555 | 1454 | 0.38 | 4 | 22 | 0.18 |
| 10 | m | 60-79 | 438976 | 6579 | 1.50 | 6261 | 1.43 | 202 | 1454 | 0.14 | 7 | 22 | 0.32 |
| 10 | m | 80+ | 108937 | 29480 | 27.06 | 28933 | 26.56 | 40 | 1454 | 0.03 | 11 | 22 | 0.50 |
| 11 | f | 15-34 | 445412 | 10331 | 2.32 | 9729 | 2.18 | 524 | 1863 | 0.28 | 0 | 17 | 0.00 |
| 11 | f | 35-59 | 709015 | 26850 | 3.79 | 25329 | 3.57 | 688 | 1863 | 0.37 | 2 | 17 | 0.12 |
| 11 | f | 60-79 | 475567 | 10941 | 2.30 | 10488 | 2.21 | 248 | 1863 | 0.13 | 5 | 17 | 0.29 |
| 11 | f | 80+ | 173095 | 52449 | 30.30 | 46493 | 26.86 | 87 | 1863 | 0.05 | 10 | 17 | 0.59 |
| 11 | m | 15-34 | 482066 | 5439 | 1.13 | 5199 | 1.08 | 551 | 1820 | 0.30 | 0 | 29 | 0.00 |
| 11 | m | 35-59 | 709197 | 10234 | 1.44 | 9714 | 1.37 | 684 | 1820 | 0.38 | 4 | 29 | 0.14 |
| 11 | m | 60-79 | 438976 | 6811 | 1.55 | 6489 | 1.48 | 217 | 1820 | 0.12 | 11 | 29 | 0.38 |
| 11 | m | 80+ | 108937 | 33699 | 30.93 | 29244 | 26.84 | 57 | 1820 | 0.03 | 14 | 29 | 0.48 |
| 12 | f | 15-34 | 445412 | 10503 | 2.36 | 9980 | 2.24 | 669 | 2354 | 0.28 | 1 | 23 | 0.04 |
| 12 | f | 35-59 | 709015 | 27472 | 3.87 | 25721 | 3.63 | 888 | 2354 | 0.38 | 0 | 23 | 0.00 |
| 12 | f | 60-79 | 475567 | 11400 | 2.40 | 10593 | 2.23 | 296 | 2354 | 0.13 | 6 | 23 | 0.26 |
| 12 | f | 80+ | 173095 | 57724 | 33.35 | 46861 | 27.07 | 100 | 2354 | 0.04 | 16 | 23 | 0.70 |

*(continued)*

| Week | Sex | Age | Population | Vaccinated | Vaccinated Percent | Vaccinated Lag 14 | Vaccinated Lag 14 Percent | SARS-CoV-2 Incidence | SARS-CoV-2 Incidence all | SARS-CoV-2 Incidence share | COVID-19 Fatality | COVID-19 Fatality all | COVID-19 Fatality share |
|---|---|---|---|---|---|---|---|---|---|---|---|---|---|
| 12 | m | 15-34 | 482066 | 5513 | 1.14 | 5311 | 1.10 | 720 | 2418 | 0.30 | 0 | 28 | 0.00 |
| 12 | m | 35-59 | 709197 | 10446 | 1.47 | 9910 | 1.40 | 924 | 2418 | 0.38 | 6 | 28 | 0.21 |
| 12 | m | 60-79 | 438976 | 7291 | 1.66 | 6579 | 1.50 | 301 | 2418 | 0.12 | 7 | 28 | 0.25 |
| 12 | m | 80+ | 108937 | 37519 | 34.44 | 29480 | 27.06 | 59 | 2418 | 0.02 | 15 | 28 | 0.54 |
| 13 | f | 15-34 | 445412 | 10642 | 2.39 | 10331 | 2.32 | 670 | 2150 | 0.31 | 0 | 31 | 0.00 |
| 13 | f | 35-59 | 709015 | 27938 | 3.94 | 26850 | 3.79 | 803 | 2150 | 0.37 | 1 | 31 | 0.03 |
| 13 | f | 60-79 | 475567 | 11771 | 2.48 | 10941 | 2.30 | 263 | 2150 | 0.12 | 6 | 31 | 0.19 |
| 13 | f | 80+ | 173095 | 68853 | 39.78 | 52449 | 30.30 | 84 | 2150 | 0.04 | 24 | 31 | 0.77 |
| 13 | m | 15-34 | 482066 | 5599 | 1.16 | 5439 | 1.13 | 725 | 2319 | 0.31 | 0 | 27 | 0.00 |
| 13 | m | 35-59 | 709197 | 10606 | 1.50 | 10234 | 1.44 | 902 | 2319 | 0.39 | 3 | 27 | 0.11 |
| 13 | m | 60-79 | 438976 | 7563 | 1.72 | 6811 | 1.55 | 314 | 2319 | 0.14 | 9 | 27 | 0.33 |
| 13 | m | 80+ | 108937 | 45770 | 42.02 | 33699 | 30.93 | 62 | 2319 | 0.03 | 15 | 27 | 0.56 |
| 14 | f | 15-34 | 445412 | 10964 | 2.46 | 10503 | 2.36 | 785 | 2502 | 0.31 | 0 | 22 | 0.00 |
| 14 | f | 35-59 | 709015 | 28804 | 4.06 | 27472 | 3.87 | 908 | 2502 | 0.36 | 3 | 22 | 0.14 |
| 14 | f | 60-79 | 475567 | 12573 | 2.64 | 11400 | 2.40 | 343 | 2502 | 0.14 | 6 | 22 | 0.27 |
| 14 | f | 80+ | 173095 | 80729 | 46.64 | 57724 | 33.35 | 81 | 2502 | 0.03 | 13 | 22 | 0.59 |
| 14 | m | 15-34 | 482066 | 5753 | 1.19 | 5513 | 1.14 | 857 | 2619 | 0.33 | 2 | 38 | 0.05 |
| 14 | m | 35-59 | 709197 | 10926 | 1.54 | 10446 | 1.47 | 930 | 2619 | 0.36 | 6 | 38 | 0.16 |
| 14 | m | 60-79 | 438976 | 8138 | 1.85 | 7291 | 1.66 | 360 | 2619 | 0.14 | 14 | 38 | 0.37 |
| 14 | m | 80+ | 108937 | 53994 | 49.56 | 37519 | 34.44 | 53 | 2619 | 0.02 | 16 | 38 | 0.42 |
| 15 | f | 15-34 | 445412 | 12808 | 2.88 | 10642 | 2.39 | 927 | 3073 | 0.30 | 1 | 29 | 0.03 |
| 15 | f | 35-59 | 709015 | 33941 | 4.79 | 27938 | 3.94 | 1123 | 3073 | 0.37 | 4 | 29 | 0.14 |
| 15 | f | 60-79 | 475567 | 17866 | 3.76 | 11771 | 2.48 | 355 | 3073 | 0.12 | 10 | 29 | 0.34 |
| 15 | f | 80+ | 173095 | 93354 | 53.93 | 68853 | 39.78 | 106 | 3073 | 0.03 | 14 | 29 | 0.48 |
| 15 | m | 15-34 | 482066 | 6808 | 1.41 | 5599 | 1.16 | 985 | 3085 | 0.32 | 0 | 36 | 0.00 |
| 15 | m | 35-59 | 709197 | 13774 | 1.94 | 10606 | 1.50 | 1118 | 3085 | 0.36 | 5 | 36 | 0.14 |
| 15 | m | 60-79 | 438976 | 12586 | 2.87 | 7563 | 1.72 | 361 | 3085 | 0.12 | 17 | 36 | 0.47 |
| 15 | m | 80+ | 108937 | 62691 | 57.55 | 45770 | 42.02 | 74 | 3085 | 0.02 | 14 | 36 | 0.39 |
| 16 | f | 15-34 | 445412 | 15525 | 3.49 | 10964 | 2.46 | 865 | 2806 | 0.31 | 0 | 22 | 0.00 |
| 16 | f | 35-59 | 709015 | 41687 | 5.88 | 28804 | 4.06 | 1020 | 2806 | 0.36 | 0 | 22 | 0.00 |
| 16 | f | 60-79 | 475567 | 21538 | 4.53 | 12573 | 2.64 | 301 | 2806 | 0.11 | 4 | 22 | 0.18 |
| 16 | f | 80+ | 173095 | 104925 | 60.62 | 80729 | 46.64 | 86 | 2806 | 0.03 | 18 | 22 | 0.82 |

*(continued)*

| Week | Sex | Age | Population | Vaccinated | Vaccinated Percent | Vaccinated Lag 14 | Vaccinated Lag 14 Percent | SARS-CoV-2 Incidence | SARS-CoV-2 Incidence all | SARS-CoV-2 Incidence share | COVID-19 Fatality | COVID-19 Fatality all | COVID-19 Fatality share |
|---|---|---|---|---|---|---|---|---|---|---|---|---|---|
| 16 | m | 15-34 | 482066 | 8165 | 1.69 | 5753 | 1.19 | 933 | 3115 | 0.30 | 0 | 31 | 0.00 |
| 16 | m | 35-59 | 709197 | 16888 | 2.38 | 10926 | 1.54 | 1147 | 3115 | 0.37 | 6 | 31 | 0.19 |
| 16 | m | 60-79 | 438976 | 15553 | 3.54 | 8138 | 1.85 | 370 | 3115 | 0.12 | 16 | 31 | 0.52 |
| 16 | m | 80+ | 108937 | 70408 | 64.63 | 53994 | 49.56 | 55 | 3115 | 0.02 | 9 | 31 | 0.29 |
| 17 | f | 15-34 | 445412 | 20232 | 4.54 | 12808 | 2.88 | 716 | 2299 | 0.31 | 0 | 18 | 0.00 |
| 17 | f | 35-59 | 709015 | 54730 | 7.72 | 33941 | 4.79 | 849 | 2299 | 0.37 | 3 | 18 | 0.17 |
| 17 | f | 60-79 | 475567 | 29890 | 6.29 | 17866 | 3.76 | 270 | 2299 | 0.12 | 5 | 18 | 0.28 |
| 17 | f | 80+ | 173095 | 118209 | 68.29 | 93354 | 53.93 | 59 | 2299 | 0.03 | 10 | 18 | 0.56 |
| 17 | m | 15-34 | 482066 | 10536 | 2.19 | 6808 | 1.41 | 809 | 2489 | 0.33 | 0 | 23 | 0.00 |
| 17 | m | 35-59 | 709197 | 23029 | 3.25 | 13774 | 1.94 | 903 | 2489 | 0.36 | 4 | 23 | 0.17 |
| 17 | m | 60-79 | 438976 | 22086 | 5.03 | 12586 | 2.87 | 308 | 2489 | 0.12 | 13 | 23 | 0.57 |
| 17 | m | 80+ | 108937 | 79178 | 72.68 | 62691 | 57.55 | 41 | 2489 | 0.02 | 6 | 23 | 0.26 |
| 18 | f | 15-34 | 445412 | 28538 | 6.41 | 15525 | 3.49 | 531 | 1818 | 0.29 | 0 | 18 | 0.00 |
| 18 | f | 35-59 | 709015 | 76998 | 10.86 | 41687 | 5.88 | 661 | 1818 | 0.36 | 2 | 18 | 0.11 |
| 18 | f | 60-79 | 475567 | 46764 | 9.83 | 21538 | 4.53 | 211 | 1818 | 0.12 | 8 | 18 | 0.44 |
| 18 | f | 80+ | 173095 | 127425 | 73.62 | 104925 | 60.62 | 50 | 1818 | 0.03 | 8 | 18 | 0.44 |
| 18 | m | 15-34 | 482066 | 14185 | 2.94 | 8165 | 1.69 | 649 | 1959 | 0.33 | 1 | 19 | 0.05 |
| 18 | m | 35-59 | 709197 | 32358 | 4.56 | 16888 | 2.38 | 702 | 1959 | 0.36 | 2 | 19 | 0.11 |
| 18 | m | 60-79 | 438976 | 35993 | 8.20 | 15553 | 3.54 | 214 | 1959 | 0.11 | 13 | 19 | 0.68 |
| 18 | m | 80+ | 108937 | 85438 | 78.43 | 70408 | 64.63 | 37 | 1959 | 0.02 | 3 | 19 | 0.16 |
| 19 | f | 15-34 | 445412 | 35578 | 7.99 | 20232 | 4.54 | 426 | 1358 | 0.31 | 0 | 5 | 0.00 |
| 19 | f | 35-59 | 709015 | 97268 | 13.72 | 54730 | 7.72 | 486 | 1358 | 0.36 | 1 | 5 | 0.20 |
| 19 | f | 60-79 | 475567 | 56194 | 11.82 | 29890 | 6.29 | 125 | 1358 | 0.09 | 3 | 5 | 0.60 |
| 19 | f | 80+ | 173095 | 129398 | 74.76 | 118209 | 68.29 | 29 | 1358 | 0.02 | 1 | 5 | 0.20 |
| 19 | m | 15-34 | 482066 | 17323 | 3.59 | 10536 | 2.19 | 451 | 1411 | 0.32 | 0 | 8 | 0.00 |
| 19 | m | 35-59 | 709197 | 40253 | 5.68 | 23029 | 3.25 | 504 | 1411 | 0.36 | 0 | 8 | 0.00 |
| 19 | m | 60-79 | 438976 | 43315 | 9.87 | 22086 | 5.03 | 145 | 1411 | 0.10 | 2 | 8 | 0.25 |
| 19 | m | 80+ | 108937 | 86723 | 79.61 | 79178 | 72.68 | 16 | 1411 | 0.01 | 6 | 8 | 0.75 |
| 20 | f | 15-34 | 445412 | 40794 | 9.16 | 28538 | 6.41 | 285 | 924 | 0.31 | 0 | 5 | 0.00 |
| 20 | f | 35-59 | 709015 | 109656 | 15.47 | 76998 | 10.86 | 293 | 924 | 0.32 | 0 | 5 | 0.00 |
| 20 | f | 60-79 | 475567 | 76124 | 16.01 | 46764 | 9.83 | 89 | 924 | 0.10 | 4 | 5 | 0.80 |
| 20 | f | 80+ | 173095 | 130003 | 75.11 | 127425 | 73.62 | 15 | 924 | 0.02 | 1 | 5 | 0.20 |

*(continued)*

| Week | Sex | Age | Population | Vaccinated | Vaccinated Percent | Vaccinated Lag 14 | Vaccinated Lag 14 Percent | SARS-CoV-2 Incidence | SARS-CoV-2 Incidence all | SARS-CoV-2 Incidence share | COVID-19 Fatality | COVID-19 Fatality all | COVID-19 Fatality share |
|---|---|---|---|---|---|---|---|---|---|---|---|---|---|
| 20 | m | 15-34 | 482066 | 20859 | 4.33 | 14185 | 2.94 | 313 | 961 | 0.33 | 0 | 6 | 0.00 |
| 20 | m | 35-59 | 709197 | 48893 | 6.89 | 32358 | 4.56 | 349 | 961 | 0.36 | 2 | 6 | 0.33 |
| 20 | m | 60-79 | 438976 | 60940 | 13.88 | 35993 | 8.20 | 94 | 961 | 0.10 | 4 | 6 | 0.67 |
| 20 | m | 80+ | 108937 | 87051 | 79.91 | 85438 | 78.43 | 8 | 961 | 0.01 | 0 | 6 | 0.00 |

Table S2. Non-pharmaceutical interventions in Rhineland-Palatinate, Germany, from calendar week 1 to 20 in 2021 [1]. X: Intervention enacted.

| | W01 | W02 | W03 | W04 | W05 | W06 | W07 | W08 | W09 | W10 | W11 | W12 | W13 | W14 | W15 | W16 | W17 | W18 | W19 | W20 |
|---|---|---|---|---|---|---|---|---|---|---|---|---|---|---|---|---|---|---|---|---|
| Secondary schools - No restrictions | | | | | | | | | | | | | | | | | | | X | X |
| Secondary schools - Restricted opening with hygiene regulations | X | X | X | X | X | X | X | X | X | X | X | X | X | X | X | X | X | X | X | X |
| Secondary schools - Different days/weeks per class/group | | | | | | | | | | X | X | X | X | X | X | X | X | X | X | X |
| Secondary schools - Only certain age groups | | | | | | | | | X | X | X | X | X | X | X | X | X | X | X | X |
| Secondary schools - Only emergency mode for kids from certain parent groups | X | X | X | X | X | X | X | X | | X | X | X | X | X | X | X | X | X | X | X |
| Elementary school - Fully open with hygiene regulations | X | X | X | X | X | X | X | X | | | | | | | | | | | | |
| Elementary school - Restricted opening with hygiene regulations | | | | | | | | | X | X | X | X | X | X | X | X | X | X | X | X |
| Elementary school - Different days/weeks per class/group | | | | | | | | | | X | X | X | X | X | X | X | X | X | X | X |
| Elementary school - Only emergency mode for kids from certain parent groups | | | | | | | | | | X | X | X | X | X | X | X | X | X | X | X |
| Masks required in secondary school classrooms | X | X | X | X | X | X | X | X | X | X | X | X | X | X | X | X | X | X | X | X |
| Masks required in elementary school classrooms | | | | | | | | | | X | X | X | X | X | X | X | X | X | X | X |
| Masks required outside in elementary schools | X | X | X | X | X | X | X | X | X | X | X | X | X | X | X | X | X | X | X | X |

*(continued)*

| | W01 | W02 | W03 | W04 | W05 | W06 | W07 | W08 | W09 | W10 | W11 | W12 | W13 | W14 | W15 | W16 | W17 | W18 | W19 | W20 |
|---|---|---|---|---|---|---|---|---|---|---|---|---|---|---|---|---|---|---|---|---|
| Culture and education institutions - Museums open with conditions | | | | | | | | | | | | | | | | | | | X | X |
| Culture and education institutions - Fully closed | X | X | X | X | X | X | X | X | X | X | X | X | X | X | X | X | X | X | X | X |
| Masks required on public transport | X | X | X | X | X | X | X | X | X | X | X | X | X | X | X | X | X | X | X | X |
| Masks required in shops | X | X | X | X | X | X | X | X | X | X | X | X | X | X | X | X | X | X | X | X |
| Masks required in public spaces | X | X | X | X | X | X | X | X | X | X | X | X | X | X | X | X | X | X | X | X |
| Indoor public events - Fully forbidden | X | X | X | X | X | X | X | X | X | X | X | X | X | X | X | X | X | X | X | X |
| Outdoor public events - Fully forbidden | X | X | X | X | X | X | X | X | X | X | X | X | X | X | X | X | X | X | X | X |
| Contact and gathering restrictions in private spaces - Recommendation to avoid social contact | X | X | X | X | X | X | X | X | X | X | X | X | X | X | X | X | X | X | X | |
| Contact and gathering restrictions in private spaces - Max 5 people, except same household / close family | | | | | | | | | | | | | | | | | | X | X | |
| Contact and gathering restrictions in private spaces - Only people from 2 households, except close family | | | | | | | | | X | X | | | | | | | | | | |
| Contact and gathering restrictions in public spaces – Recommendation to avoid social contact | | X | X | X | X | X | X | X | | | | | | | | | | X | X | X |
| Gastronomy - Restricted opening with hygiene regulations | X | X | X | X | X | X | X | X | X | X | X | X | X | X | X | X | X | X | X | X |
| Gastronomy - Only outside dining | | | | | | | | | | | | | | | | | | X | X | X |
| Gastronomy - Only delivery and pick-up | X | X | X | X | X | X | X | X | X | X | X | X | X | X | X | X | X | X | X | X |
| Small and large shops - Restricted opening with hygiene regulations | X | X | X | X | X | X | X | X | | | | | | | | | | | | |
| Small and large shops - Only critical provisions | | X | X | X | X | X | X | X | X | X | X | X | X | X | X | X | X | X | X | X |
| Small and large shops - Critical provisions including bookstores and flower shops | X | | | | | | | | | | | | | | | | | | | |
| Services and Trades - Fully open with hygiene regulations | | | | | | | | | | | | | | | | | | | X | X |
| Services and Trades - Restricted opening with hygiene regulations | X | X | X | X | X | X | X | X | X | X | X | X | X | X | X | X | X | X | X | X |

*(continued)*

| | W01 | W02 | W03 | W04 | W05 | W06 | W07 | W08 | W09 | W10 | W11 | W12 | W13 | W14 | W15 | W16 | W17 | W18 | W19 | W20 |
|---|---|---|---|---|---|---|---|---|---|---|---|---|---|---|---|---|---|---|---|---|
| Services and Trades - Only services with avoidable customer contact and health/care services | X | X | X | X | X | X | X | X | X | X | X | X | X | X | X | X | X | X | X | X |
| Services and Trades - Only by appointment | | | | | | | | | X | | | | | | | | | | | |
| Services and Trades - Entry resrtictions related to testing | | | | | | | | | | X | | | | | | | | | | |
| Social distancing - Introduction of social distancing rules | X | X | X | X | X | X | X | X | X | X | X | X | X | X | X | X | X | X | X | X |
| Testing - Required for system-relevant professions | | | | | | | | | X | X | X | X | X | X | X | X | X | X | X | X |

Table S3. COVID-19 vaccination prioritization policy in Rhineland-Palatinate, Germany, from calendar week 1 to 20 in 2021. X: Eligible for vaccination.

| Priority | W01 | W02 | W03 | W04 | W05 | W06 | W07 | W08 | W09 | W10 | W11 | W12 | W13 | W14 | W15 | W16 | W17 | W18 | W19 | W20 |
|---|---|---|---|---|---|---|---|---|---|---|---|---|---|---|---|---|---|---|---|---|
| Priority group I - Age group 80+, nursing home residents, health care workers with very high risk of exposure | X | X | X | X | X | X | X | X | X | X | X | X | X | X | X | X | X | X | X | X |
| Priority group II - Persons in certain occupational groups, with underlying medical conditions, in need of care, with contact to vulnerable persons, health care workers with high risk of exposure | | | | | | | | X | X | X | X | X | X | X | X | X | X | X | X | X |
| Age group 70-79 | | | | | | | | | | X | X | X | X | X | X | X | X | X | X | X |
| Age group 60-69 | | | | | | | | | | | | | | X | X | X | X | X | X | X |
| Priority group III - Secondary school teachers, grocery store workers, workers in critical infrastructure | | | | | | | | | | | | | | | | X | X | X | X | X |

Table S4. Absolute frequency (percentage) of vaccines used for the first shot from calendar week 1 to 20 in 2021 in Rhineland-Palatinate, Germany. NA: Not Available.

| Age group | NA | BNT15b2 | mRNA1273 | Vaxzevria | Janssen |
|---|---|---|---|---|---|
| 15-34 | 36 (0.0) | 76633 (57.6) | 21116 (15.9) | 35172 (26.4) | 175 (0.1) |
| 35-59 | 66 (0.0) | 194602 (59.7) | 47116 (14.5) | 84032 (25.8) | 129 (0.0) |
| 60-79 | 89 (0.0) | 179766 (52.4) | 28766 (8.4) | 134234 (39.1) | 22 (0.0) |
| 80+ | 17 (0.0) | 214456 (95.6) | 7192 (3.2) | 2775 (1.2) | 1 (0.0) |

Table S5. Absolute frequency (percentage) of vaccines used for the second shot from calendar week 1 to 20 in 2021 in Rhineland-Palatinate, Germany.

| Age group | BNT15b2 | mRNA1273 | Vaxzevria |
|---|---|---|---|
| 15-34 | 46057 (57.6) | 6499 (15.9) | 5856 (26.4) |
| 35-59 | 117301 (59.7) | 16164 (14.5) | 19455 (25.8) |
| 60-79 | 116933 (52.4) | 11140 (8.4) | 7632 (39.1) |
| 80+ | 210023 (95.6) | 6539 (3.2) | 31 (1.2) |

Table S6. Adjusted odds ratios (OR), 95% confidence intervals (CI) and p-values (p) from the multivariable logistic regression model for the sex-specific proportion of all-cause mortality formed by each age group.

| Variable | OR | CI | p |
|---|---|---|---|
| Sex male | 1.003 | [0.876, 1.147] | 0.97 |
| Age 15-34 | 0.004 | [0.003, 0.006] | 0.00 |
| Age 35-59 | 0.052 | [0.041, 0.066] | 0.00 |
| Age 60-79 | 0.292 | [0.226, 0.378] | 0.00 |
| Proportion vaccinated | 0.996 | [0.991, 1.001] | 0.16 |

# 11 References


1. German Federal Statistical Office. Sterbefälle - Fallzahlen nach Tagen, Wochen, Monaten, Altersgruppen, Geschlecht und Bundesländern für Deutschland 2016 - 2021 [Internet]. 2021 [cited 2021 Nov 18]. Available from: https://www.destatis.de/DE/Themen/Gesellschaft-Umwelt/Bevoelkerung/Sterbefaelle-Lebenserwartung/sterbefallzahlen.html

2. Pezzullo AM, Villani L, Causio FA, Axfors C, Contopoulos-Ioannidis DG, Boccia S, Ioannidis JPA. Change in age distribution of COVID-19 deaths with the introduction of COVID-19 vaccination. Environ Res. 2021 Nov 5:112342. doi: 10.1016/j.envres.2021.112342.

3. Corona Datenplattform. [Internet]. 2021 [cited 2021 Nov 18]. Available from: https://www.corona-datenplattform.de/

4. Steiger E, Rass S, Seidel A, Kroll L, Czihal T. COVID-19 vaccination in medical practices in Germany. Dtsch Arztebl Int 2021; 118: 756-7; DOI: 10.3238/arztebl.m2021.0354

5. Robert Koch Institute. Tabellen zu Testzahlen, Testkapazitäten und Probenrückstau pro Woche [Internet]. 2021 [cited 2021 Nov 18]. Available from: https://www.rki.de/DE/Content/InfAZ/N/Neuartiges_Coronavirus/Daten/Testzahlen-gesamt.html

6. Robert Koch Institute. RKI COVID19 Dashboard Data [Internet]. 2021 [cited 2021 Nov 18]. Open Data license Germany - attribution - Version 2.0: dl-de/by-2-0. Available from: https://npgeo-corona-npgeo-de.hub.arcgis.com/datasets/dd4580c810204019a7b8eb3e0b329dd6_0/data
